# (4′-All­yloxy-2,2′:6′,2′′-terpyridine-κ^3^ *N*,*N*′,*N*′′)(dibenzoyl­methanido-κ^2^ *O*,*O*′)bis­(nitrato-κ^2^ *O*,*O*′)neodymium(III) acetonitrile solvate

**DOI:** 10.1107/S160053680905185X

**Published:** 2009-12-04

**Authors:** Qunbo Mei, Bihai Tong

**Affiliations:** a Jiangsu Key Laboratory of Organic Electronics and Information Displays, and Institute of Advanced Materials (IAM), Nanjing University of Post and Telecommunications, Nanjing 210046, People’s Republic of China; b Institute of Molecular Engineering and Applied Chemistry, College of Metallurgy and Resources, Anhui University of Technology, Maanshan 243002, People’s Republic of China

## Abstract

The title complex, [Nd(C_15_H_11_O_2_)(NO_3_)_2_(C_18_H_15_N_3_O)]·CH_3_CN or [Nd(altpy)(dbm)(NO_3_)_2_]·CH_3_CN (altpy = 4′-all­yl­oxy-2,2′:6′,2′′-terpyridine, dbm = dibenzoyl­methanide anion), has been synthesized from 4′-all­yloxy-2,2′:6′,2′′-terpyridine, dibenzoyl­methanate and neodymium nitrate. The Nd^3+^ atom is nine-coordinated by two O atoms from the bidentate dbm ligand, three N atoms from the tridentate altpy ligand and four O atoms from two nitrate anions that act as bidentate ligands and occupy mutually *trans* sites in a distorted monocapped square-anti­prismatic geometry.

## Related literature

For the use of lanthanide complexes as shift reagents, functional materials and as catalysts, see: Su *et al.* (1999 ▶); Sutter *et al.* (1998 ▶); Aspinall *et al.* (1998 ▶). For related structures, see: Niu *et al.* (1997 ▶); Chen *et al.* (1998 ▶); Cotton *et al.* (2003 ▶); Hunter *et al.* (2007 ▶). 
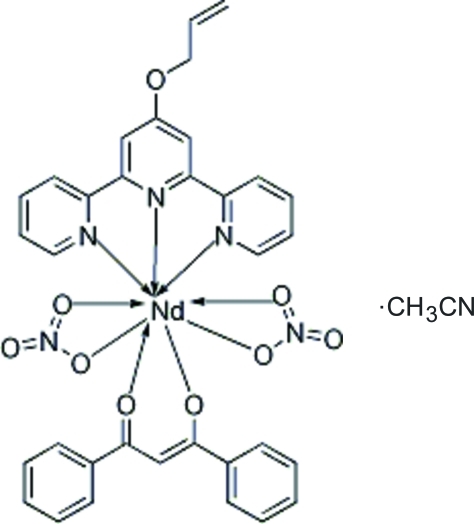


## Experimental

### 

#### Crystal data

[Nd(C_15_H_11_O_2_)(NO_3_)_2_(C_18_H_15_N_3_O)]·C_2_H_3_N
*M* *_r_* = 821.88
Monoclinic, 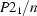

*a* = 13.3711 (16) Å
*b* = 16.1009 (19) Å
*c* = 15.9490 (19) Å
β = 103.040 (2)°
*V* = 3345.1 (7) Å^3^
*Z* = 4
Mo *K*α radiation
μ = 1.62 mm^−1^
*T* = 569 K
0.41 × 0.36 × 0.25 mm

#### Data collection

Bruker SMART CCD area-detector diffractometer
Absorption correction: multi-scan *SADABS* (Bruker, 1997 ▶) *T*_min_ = 0.557, *T*_max_ = 0.688
6565 measured reflections
6565 independent reflections
5101 reflections with *I* > 2σ(*I*)

#### Refinement

*R*[*F* ^2^ > 2σ(*F* ^2^)] = 0.029
*wR*(*F* ^2^) = 0.069
*S* = 1.09
6565 reflections
461 parameters
H-atom parameters constrained
Δρ_max_ = 0.91 e Å^−3^
Δρ_min_ = −0.46 e Å^−3^

### 

Data collection: *SMART* (Bruker, 1997 ▶); cell refinement: *SAINT* (Bruker, 1997 ▶); data reduction: *SAINT*; program(s) used to solve structure: *SHELXS97* (Sheldrick, 2008 ▶); program(s) used to refine structure: *SHELXL97* (Sheldrick, 2008 ▶); molecular graphics: *SHELXTL* (Sheldrick, 2008 ▶); software used to prepare material for publication: *SHELXTL*.

## Supplementary Material

Crystal structure: contains datablocks I, global. DOI: 10.1107/S160053680905185X/sj2697sup1.cif

Structure factors: contains datablocks I. DOI: 10.1107/S160053680905185X/sj2697Isup2.hkl

Additional supplementary materials:  crystallographic information; 3D view; checkCIF report

## Figures and Tables

**Table 1:** Selected bond lengths (Å)

Nd1—O2	2.343 (2)
Nd1—O3	2.354 (2)
Nd1—O6	2.550 (2)
Nd1—O9	2.558 (2)
Nd1—O8	2.559 (2)
Nd1—O5	2.571 (3)
Nd1—N3	2.578 (3)
Nd1—N1	2.603 (2)
Nd1—N2	2.605 (2)

**Fig. 1.**
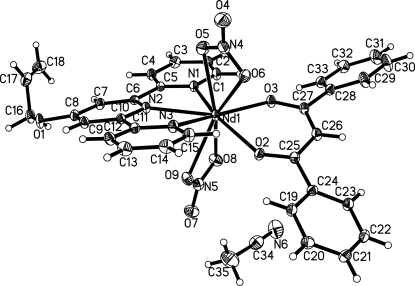
The molecular structure of (I), with atom labels and 30% probability displacement ellipsoids for non-H atoms.

## supplementary crystallographic information

### Comment

Lanthanide complexes are often used as shift reagents to probe metal-binding sites in solutions (Su *et al.*, 1999) and they are also used in functional materials (Sutter *et al.*, 1998) or catalysts (Aspinall *et al.*, 1998). The ability of these elements to adopt coordination numbers from six to twelve provides a rich and variable structural chemistry. In the title compound, [Nd(altpy)(dbm)(NO_3_)_2_].CH_3_CN(altpy=4'-allyloxy-2, 2':6', 2''-terpyridine, dbm=dibenzoylmethanate), each Nd(III) atom is in a nine coordinate environment comprising two oxygen atoms from the bidentate dbm ligand, three nitrogen atoms from the tridentate altpy ligand and four oxygen atoms from two tertiary nitrate anions that act as bidentate ligands an occupy mutually *trans* sites in the coordination polyhedron. The coordination polyhedron is a distorted monocapped square antiprism. The Nd—O distances lie in two groups, those to the beta-diketone oxygen atoms in the range 2.343 (2)–2.354 (2) Å and those to nitrate O atoms in the range 2.550 (3)–2.573 (3) Å. These are comparable to those [2.485 (19), 2.600 (15) Å] in the nine-coordinate complex [Nd_2_(O_2_CMe)_4_(NO_3_)_2_(phen)_2_] (phen=1,10-phenanthroline) which also contains bidentate chelating nitrate anions (Niu *et al.*, 1997). The O—Nd—O angle (73.64 (8) °) of the beta-diketonate ligand is somewhat higher as compared to those found in the neodymium tris(beta-diketonates) type of complexes (Chen *et al.*, 1998). The average Nd—N distance (2.595 (3) Å) is slightly longer than that in the nine-coordinate complex [Eu(terpy)(NO_3_)_3_^-^(H_2_O)] (2.554 Å)(Cotton *et al.* (2003)).The geometrical parameters of the [NO_3_]^-^ anions in the title complex are as expected with normal distances and angles, comparable to those reported by Hunter *et al.*, (2007) for a complex also containing bidentate chelating nitrate anions

### Experimental

The title compound was obtained by refluxing neodymium nitrate, 4'-allyloxy-2, 2':6',2''-terpyridine and dibenzoylmethanate in ethanol to give the title compound as a blue precipitate in 78% yield. Recrystallization from ethanol and acetonitrile (1:1) gave blue block-like crystals suitable for an X-ray diffraction determination. Anal.Calcd. for C_35_H_30_N_6_NdO_9_: C, 51.03, H, 3.64, N, 10.21%. Found:*C*, 51.10, H, 3.67, N, 10.12%.

### Refinement

H atoms were positioned geometrically and refined using a riding model (including free rotation about the ethanol C—C bond), with C—H = 0.95–0.99 Å and with *U*_iso_(H) = 1.2 (1.5 for methyl groups) *U*_eq_(C).

### Crystal data

**Table:**

[Nd(C_15_H_11_O_2_)(NO_3_)_2_(C_18_H_15_N_3_O)]·C_2_H_3_N	*F*(000) = 1652
*M**_r_* = 821.88	*D*_x_ = 1.632 Mg m^−^^3^
Monoclinic, *P*2_1_/*n*	Mo *K*α radiation, λ = 0.71073 Å
Hall symbol: -P 2yn	Cell parameters from 4901 reflections
*a* = 13.3711 (16) Å	θ = 2.2–27.1°
*b* = 16.1009 (19) Å	µ = 1.62 mm^−^^1^
*c* = 15.9490 (19) Å	*T* = 569 K
β = 103.040 (2)°	Block, blue
*V* = 3345.1 (7) Å^3^	0.41 × 0.36 × 0.25 mm
*Z* = 4	

### Data collection

**Table:**

Bruker SMART CCD area-detector diffractometer	6565 independent reflections
Radiation source: fine-focus sealed tube	5101 reflections with *I* > 2σ(*I*)
graphite	*R*_int_ = 0.0000
φ and ω scans	θ_max_ = 26.0°, θ_min_ = 1.8°
Absorption correction: multi-scan *SADABS* (Bruker, 1997)	*h* = −16→16
*T*_min_ = 0.557, *T*_max_ = 0.688	*k* = 0→19
6565 measured reflections	*l* = 0→19

### Refinement

**Table:**

Refinement on *F*^2^	Primary atom site location: structure-invariant direct methods
Least-squares matrix: full	Secondary atom site location: difference Fourier map
*R*[*F*^2^ > 2σ(*F*^2^)] = 0.029	Hydrogen site location: inferred from neighbouring sites
*wR*(*F*^2^) = 0.069	H-atom parameters constrained
*S* = 1.09	*w* = 1/[σ^2^(*F*_o_^2^) + (0.0298*P*)^2^ + 2.1769*P*] where *P* = (*F*_o_^2^ + 2*F*_c_^2^)/3
6565 reflections	(Δ/σ)_max_ = 0.002
461 parameters	Δρ_max_ = 0.91 e Å^−^^3^
0 restraints	Δρ_min_ = −0.46 e Å^−^^3^

### Special details

**Table:**

Geometry. All e.s.d.'s (except the e.s.d. in the dihedral angle between two l.s. planes) are estimated using the full covariance matrix. The cell e.s.d.'s are taken into account individually in the estimation of e.s.d.'s in distances, angles and torsion angles; correlations between e.s.d.'s in cell parameters are only used when they are defined by crystal symmetry. An approximate (isotropic) treatment of cell e.s.d.'s is used for estimating e.s.d.'s involving l.s. planes.
Refinement. Refinement of *F*^2^ against ALL reflections. The weighted *R*-factor *wR* and goodness of fit *S* are based on *F*^2^, conventional *R*-factors *R* are based on *F*, with *F* set to zero for negative *F*^2^. The threshold expression of *F*^2^ > σ(*F*^2^) is used only for calculating *R*-factors(gt) *etc*. and is not relevant to the choice of reflections for refinement. *R*-factors based on *F*^2^ are statistically about twice as large as those based on *F*, and *R*- factors based on ALL data will be even larger.

### Fractional atomic coordinates and isotropic or equivalent isotropic displacement parameters (Å^2^)

**Table:**

	*x*	*y*	*z*	*U*_iso_*/*U*_eq_	
Nd1	0.754510 (13)	0.201262 (9)	0.532547 (11)	0.02286 (6)	
O1	0.65947 (18)	−0.20624 (13)	0.48248 (16)	0.0353 (6)	
O2	0.66289 (17)	0.31699 (12)	0.56350 (14)	0.0284 (5)	
O3	0.87760 (16)	0.30266 (12)	0.59043 (15)	0.0297 (5)	
O4	0.7676 (2)	0.26540 (18)	0.28068 (18)	0.0557 (8)	
O5	0.8192 (2)	0.18627 (14)	0.39321 (17)	0.0406 (6)	
O6	0.73546 (19)	0.29852 (13)	0.40375 (16)	0.0359 (6)	
O7	0.6621 (2)	0.11468 (16)	0.75275 (16)	0.0447 (7)	
O8	0.78009 (18)	0.16492 (16)	0.69217 (15)	0.0381 (6)	
O9	0.62830 (17)	0.14135 (14)	0.61564 (15)	0.0343 (6)	
N1	0.91391 (19)	0.10513 (15)	0.57890 (17)	0.0253 (6)	
N2	0.73078 (19)	0.04405 (15)	0.49459 (17)	0.0226 (6)	
N3	0.5878 (2)	0.15850 (15)	0.42526 (17)	0.0272 (6)	
N4	0.7744 (2)	0.25116 (18)	0.3572 (2)	0.0365 (7)	
N5	0.6896 (2)	0.13977 (17)	0.68938 (19)	0.0307 (6)	
C1	1.0065 (2)	0.1370 (2)	0.6148 (2)	0.0303 (8)	
H1A	1.0126	0.1945	0.6190	0.036*	
C2	1.0924 (3)	0.0903 (2)	0.6457 (2)	0.0316 (8)	
H2A	1.1550	0.1154	0.6694	0.038*	
C3	1.0836 (3)	0.0049 (2)	0.6407 (2)	0.0307 (8)	
H3A	1.1403	−0.0289	0.6610	0.037*	
C4	0.9891 (2)	−0.02924 (19)	0.6049 (2)	0.0292 (7)	
H4A	0.9817	−0.0867	0.6015	0.035*	
C5	0.9050 (2)	0.02128 (18)	0.57400 (19)	0.0217 (7)	
C6	0.8018 (2)	−0.01195 (18)	0.53183 (19)	0.0221 (7)	
C7	0.7835 (2)	−0.09649 (19)	0.5309 (2)	0.0266 (7)	
H7A	0.8338	−0.1331	0.5590	0.032*	
C8	0.6886 (3)	−0.12537 (18)	0.4872 (2)	0.0273 (7)	
C9	0.6162 (2)	−0.06896 (19)	0.4457 (2)	0.0268 (7)	
H9A	0.5529	−0.0873	0.4143	0.032*	
C10	0.6386 (2)	0.01495 (18)	0.45141 (19)	0.0221 (7)	
C11	0.5613 (2)	0.07775 (19)	0.4104 (2)	0.0241 (7)	
C12	0.4668 (2)	0.0560 (2)	0.3595 (2)	0.0306 (8)	
H12A	0.4500	0.0003	0.3488	0.037*	
C13	0.3977 (3)	0.1170 (2)	0.3246 (2)	0.0345 (8)	
H13A	0.3332	0.1029	0.2918	0.041*	
C14	0.4251 (3)	0.1991 (2)	0.3389 (2)	0.0344 (8)	
H14A	0.3804	0.2414	0.3148	0.041*	
C15	0.5201 (2)	0.2169 (2)	0.3894 (2)	0.0312 (8)	
H15A	0.5385	0.2724	0.3994	0.037*	
C16	0.7296 (3)	−0.2675 (2)	0.5289 (2)	0.0360 (9)	
H16A	0.6917	−0.3175	0.5357	0.043*	
H16B	0.7602	−0.2464	0.5859	0.043*	
C17	0.8127 (3)	−0.2887 (2)	0.4840 (3)	0.0404 (9)	
H17A	0.7947	−0.2985	0.4251	0.048*	
C18	0.9096 (3)	−0.2942 (2)	0.5230 (3)	0.0482 (10)	
H18A	0.9297	−0.2846	0.5819	0.058*	
H18B	0.9583	−0.3076	0.4919	0.058*	
C19	0.4968 (2)	0.4175 (2)	0.5694 (2)	0.0303 (8)	
H19A	0.4850	0.3694	0.5364	0.036*	
C20	0.4149 (3)	0.4633 (2)	0.5825 (2)	0.0348 (8)	
H20A	0.3482	0.4463	0.5579	0.042*	
C21	0.4313 (3)	0.5348 (2)	0.6323 (2)	0.0354 (8)	
H21A	0.3761	0.5662	0.6407	0.042*	
C22	0.5302 (3)	0.5587 (2)	0.6692 (2)	0.0330 (8)	
H22A	0.5415	0.6058	0.7038	0.040*	
C23	0.6124 (3)	0.5138 (2)	0.6556 (2)	0.0308 (8)	
H23A	0.6787	0.5312	0.6806	0.037*	
C24	0.5976 (2)	0.44244 (19)	0.6050 (2)	0.0251 (7)	
C25	0.6845 (2)	0.39060 (19)	0.5897 (2)	0.0253 (7)	
C26	0.7840 (2)	0.4243 (2)	0.6058 (2)	0.0285 (7)	
H26	0.7904	0.4809	0.6174	0.034*	
C27	0.8732 (2)	0.38068 (19)	0.6059 (2)	0.0256 (7)	
C28	0.9738 (2)	0.4246 (2)	0.6312 (2)	0.0281 (7)	
C29	0.9844 (3)	0.5074 (2)	0.6094 (3)	0.0413 (9)	
H29A	0.9280	0.5365	0.5785	0.050*	
C30	1.0788 (3)	0.5464 (2)	0.6338 (3)	0.0544 (12)	
H30A	1.0857	0.6012	0.6176	0.065*	
C31	1.1621 (3)	0.5051 (2)	0.6814 (3)	0.0474 (10)	
H31A	1.2248	0.5323	0.6985	0.057*	
C32	1.1530 (3)	0.4234 (2)	0.7041 (2)	0.0378 (9)	
H32A	1.2093	0.3953	0.7368	0.045*	
C33	1.0595 (2)	0.3832 (2)	0.6780 (2)	0.0304 (8)	
H33A	1.0540	0.3276	0.6919	0.036*	
N6	0.6450 (3)	0.3229 (2)	0.7792 (2)	0.0617 (10)	
C34	0.5672 (3)	0.2950 (2)	0.7485 (2)	0.0437 (9)	
C35	0.4712 (3)	0.2565 (3)	0.7093 (3)	0.0672 (14)	
H35A	0.4633	0.2553	0.6480	0.101*	
H35B	0.4159	0.2876	0.7233	0.101*	
H35C	0.4701	0.2007	0.7305	0.101*	

### Atomic displacement parameters (Å^2^)

**Table:**

	*U*^11^	*U*^22^	*U*^33^	*U*^12^	*U*^13^	*U*^23^
Nd1	0.01860 (9)	0.01540 (8)	0.03276 (10)	−0.00015 (7)	0.00199 (7)	−0.00221 (8)
O1	0.0337 (13)	0.0161 (11)	0.0516 (15)	−0.0058 (10)	0.0004 (11)	−0.0030 (11)
O2	0.0249 (12)	0.0218 (11)	0.0377 (13)	0.0013 (9)	0.0056 (10)	−0.0055 (10)
O3	0.0175 (11)	0.0207 (11)	0.0493 (14)	−0.0011 (9)	0.0042 (10)	−0.0045 (11)
O4	0.077 (2)	0.0503 (17)	0.0436 (17)	0.0050 (16)	0.0222 (16)	0.0098 (14)
O5	0.0416 (15)	0.0335 (14)	0.0478 (15)	0.0110 (11)	0.0128 (12)	0.0039 (12)
O6	0.0406 (14)	0.0231 (11)	0.0472 (14)	0.0069 (11)	0.0168 (12)	0.0005 (11)
O7	0.0451 (16)	0.0499 (16)	0.0405 (15)	0.0039 (13)	0.0128 (13)	0.0123 (13)
O8	0.0231 (13)	0.0509 (15)	0.0372 (14)	−0.0033 (12)	−0.0001 (11)	0.0041 (12)
O9	0.0273 (13)	0.0358 (13)	0.0363 (14)	−0.0086 (11)	0.0000 (11)	−0.0002 (11)
N1	0.0224 (14)	0.0195 (13)	0.0330 (15)	−0.0005 (11)	0.0038 (12)	−0.0016 (12)
N2	0.0212 (14)	0.0163 (12)	0.0287 (14)	−0.0015 (11)	0.0022 (11)	−0.0001 (11)
N3	0.0249 (15)	0.0216 (14)	0.0329 (16)	0.0029 (12)	0.0022 (12)	0.0002 (12)
N4	0.0354 (18)	0.0287 (17)	0.046 (2)	−0.0041 (14)	0.0112 (15)	0.0031 (14)
N5	0.0266 (16)	0.0260 (14)	0.0378 (18)	0.0053 (12)	0.0035 (14)	0.0025 (13)
C1	0.0252 (18)	0.0207 (16)	0.043 (2)	−0.0014 (14)	0.0024 (16)	−0.0021 (15)
C2	0.0236 (18)	0.0329 (18)	0.0362 (19)	−0.0023 (15)	0.0023 (15)	−0.0035 (16)
C3	0.0244 (18)	0.0315 (18)	0.0323 (19)	0.0083 (15)	−0.0019 (15)	0.0017 (15)
C4	0.0295 (19)	0.0203 (16)	0.0364 (19)	0.0010 (14)	0.0045 (15)	0.0007 (14)
C5	0.0232 (17)	0.0187 (15)	0.0232 (16)	−0.0002 (13)	0.0051 (13)	−0.0002 (13)
C6	0.0171 (16)	0.0218 (15)	0.0276 (17)	0.0012 (13)	0.0052 (13)	−0.0031 (13)
C7	0.0253 (17)	0.0203 (15)	0.0325 (18)	0.0027 (13)	0.0030 (15)	−0.0014 (14)
C8	0.0304 (18)	0.0180 (15)	0.0339 (19)	−0.0020 (14)	0.0081 (15)	−0.0037 (14)
C9	0.0204 (17)	0.0250 (16)	0.0330 (18)	−0.0030 (13)	0.0016 (14)	−0.0031 (14)
C10	0.0201 (16)	0.0207 (15)	0.0248 (16)	−0.0021 (13)	0.0035 (13)	−0.0019 (13)
C11	0.0224 (17)	0.0255 (16)	0.0238 (16)	−0.0017 (13)	0.0043 (13)	−0.0025 (13)
C12	0.0250 (18)	0.0301 (17)	0.0347 (19)	−0.0038 (15)	0.0026 (15)	0.0000 (15)
C13	0.0221 (18)	0.046 (2)	0.0308 (19)	0.0003 (16)	−0.0043 (15)	0.0022 (16)
C14	0.0306 (19)	0.0372 (19)	0.0336 (19)	0.0076 (17)	0.0037 (15)	0.0069 (17)
C15	0.0269 (18)	0.0276 (18)	0.0360 (19)	0.0026 (14)	0.0009 (15)	0.0003 (14)
C16	0.038 (2)	0.0177 (15)	0.050 (2)	−0.0010 (15)	0.0051 (19)	0.0008 (16)
C17	0.050 (2)	0.0289 (19)	0.039 (2)	0.0042 (17)	0.0052 (18)	−0.0038 (16)
C18	0.055 (3)	0.040 (2)	0.053 (2)	0.016 (2)	0.017 (2)	0.001 (2)
C19	0.0275 (18)	0.0242 (17)	0.039 (2)	−0.0009 (14)	0.0061 (15)	−0.0052 (15)
C20	0.0235 (18)	0.0361 (19)	0.045 (2)	0.0012 (15)	0.0073 (16)	−0.0022 (17)
C21	0.035 (2)	0.0285 (18)	0.046 (2)	0.0088 (16)	0.0145 (17)	0.0014 (16)
C22	0.037 (2)	0.0238 (17)	0.038 (2)	0.0009 (15)	0.0098 (17)	−0.0066 (15)
C23	0.0261 (18)	0.0256 (17)	0.039 (2)	0.0000 (14)	0.0037 (15)	−0.0037 (15)
C24	0.0260 (17)	0.0242 (16)	0.0257 (17)	0.0034 (14)	0.0072 (14)	0.0007 (13)
C25	0.0266 (17)	0.0226 (16)	0.0252 (17)	−0.0001 (14)	0.0024 (14)	0.0010 (13)
C26	0.0213 (17)	0.0234 (16)	0.038 (2)	−0.0002 (14)	0.0012 (15)	0.0002 (14)
C27	0.0235 (17)	0.0231 (16)	0.0272 (17)	−0.0022 (13)	−0.0003 (14)	−0.0014 (13)
C28	0.0228 (17)	0.0262 (17)	0.0346 (19)	−0.0028 (14)	0.0050 (15)	−0.0073 (14)
C29	0.027 (2)	0.0290 (18)	0.062 (3)	−0.0025 (16)	−0.0009 (18)	0.0064 (18)
C30	0.040 (2)	0.033 (2)	0.085 (3)	−0.0126 (18)	0.003 (2)	0.007 (2)
C31	0.026 (2)	0.045 (2)	0.068 (3)	−0.0131 (18)	0.0032 (19)	−0.003 (2)
C32	0.0223 (18)	0.041 (2)	0.046 (2)	0.0005 (16)	0.0007 (16)	−0.0059 (18)
C33	0.0257 (18)	0.0282 (17)	0.0362 (19)	−0.0008 (14)	0.0048 (15)	−0.0040 (15)
N6	0.067 (3)	0.061 (2)	0.047 (2)	−0.009 (2)	−0.007 (2)	0.0015 (18)
C34	0.050 (3)	0.047 (2)	0.033 (2)	0.006 (2)	0.0069 (19)	0.0003 (19)
C35	0.044 (3)	0.080 (4)	0.074 (3)	−0.006 (3)	0.006 (3)	0.007 (3)

### Geometric parameters (Å, °)

**Table:**

Nd1—O2	2.343 (2)	C12—H12A	0.9300
Nd1—O3	2.354 (2)	C13—C14	1.377 (5)
Nd1—O6	2.550 (2)	C13—H13A	0.9300
Nd1—O9	2.558 (2)	C14—C15	1.373 (5)
Nd1—O8	2.559 (2)	C14—H14A	0.9300
Nd1—O5	2.571 (3)	C15—H15A	0.9300
Nd1—N3	2.578 (3)	C16—C17	1.491 (5)
Nd1—N1	2.603 (2)	C16—H16A	0.9700
Nd1—N2	2.605 (2)	C16—H16B	0.9700
Nd1—N4	2.979 (3)	C17—C18	1.307 (5)
Nd1—N5	2.995 (3)	C17—H17A	0.9300
O1—C8	1.356 (3)	C18—H18A	0.9300
O1—C16	1.445 (4)	C18—H18B	0.9300
O2—C25	1.268 (4)	C19—C20	1.374 (5)
O3—C27	1.284 (4)	C19—C24	1.398 (4)
O4—N4	1.224 (4)	C19—H19A	0.9300
O5—N4	1.275 (4)	C20—C21	1.388 (5)
O6—N4	1.257 (4)	C20—H20A	0.9300
O7—N5	1.220 (3)	C21—C22	1.375 (5)
O8—N5	1.267 (3)	C21—H21A	0.9300
O9—N5	1.273 (3)	C22—C23	1.373 (5)
N1—C1	1.343 (4)	C22—H22A	0.9300
N1—C5	1.356 (4)	C23—C24	1.392 (4)
N2—C6	1.347 (4)	C23—H23A	0.9300
N2—C10	1.353 (4)	C24—C25	1.495 (4)
N3—C15	1.341 (4)	C25—C26	1.405 (4)
N3—C11	1.355 (4)	C26—C27	1.384 (4)
C1—C2	1.368 (4)	C26—H26	0.9300
C1—H1A	0.9300	C27—C28	1.493 (4)
C2—C3	1.382 (5)	C28—C33	1.388 (5)
C2—H2A	0.9300	C28—C29	1.392 (5)
C3—C4	1.377 (4)	C29—C30	1.384 (5)
C3—H3A	0.9300	C29—H29A	0.9300
C4—C5	1.386 (4)	C30—C31	1.371 (5)
C4—H4A	0.9300	C30—H30A	0.9300
C5—C6	1.491 (4)	C31—C32	1.377 (5)
C6—C7	1.383 (4)	C31—H31A	0.9300
C7—C8	1.383 (4)	C32—C33	1.386 (5)
C7—H7A	0.9300	C32—H32A	0.9300
C8—C9	1.382 (4)	C33—H33A	0.9300
C9—C10	1.382 (4)	N6—C34	1.138 (5)
C9—H9A	0.9300	C34—C35	1.436 (6)
C10—C11	1.488 (4)	C35—H35A	0.9600
C11—C12	1.384 (4)	C35—H35B	0.9600
C12—C13	1.377 (5)	C35—H35C	0.9600
			
O2—Nd1—O3	73.64 (7)	N1—C5—C6	116.3 (3)
O2—Nd1—O6	73.70 (7)	C4—C5—C6	123.0 (3)
O3—Nd1—O6	79.86 (8)	N2—C6—C7	123.3 (3)
O2—Nd1—O9	75.72 (7)	N2—C6—C5	116.6 (3)
O3—Nd1—O9	122.89 (8)	C7—C6—C5	120.1 (3)
O6—Nd1—O9	133.74 (8)	C6—C7—C8	118.5 (3)
O2—Nd1—O8	86.05 (8)	C6—C7—H7A	120.8
O3—Nd1—O8	81.01 (8)	C8—C7—H7A	120.8
O6—Nd1—O8	155.32 (8)	O1—C8—C9	116.3 (3)
O9—Nd1—O8	49.77 (7)	O1—C8—C7	124.7 (3)
O2—Nd1—O5	123.40 (8)	C9—C8—C7	119.0 (3)
O3—Nd1—O5	93.58 (8)	C10—C9—C8	119.5 (3)
O6—Nd1—O5	49.70 (7)	C10—C9—H9A	120.2
O9—Nd1—O5	143.33 (8)	C8—C9—H9A	120.2
O8—Nd1—O5	147.45 (8)	N2—C10—C9	122.1 (3)
O2—Nd1—N3	86.42 (8)	N2—C10—C11	116.8 (3)
O3—Nd1—N3	150.31 (8)	C9—C10—C11	121.1 (3)
O6—Nd1—N3	73.44 (8)	N3—C11—C12	121.0 (3)
O9—Nd1—N3	70.77 (8)	N3—C11—C10	116.5 (3)
O8—Nd1—N3	120.05 (8)	C12—C11—C10	122.5 (3)
O5—Nd1—N3	79.05 (8)	C13—C12—C11	119.8 (3)
O2—Nd1—N1	147.86 (8)	C13—C12—H12A	120.1
O3—Nd1—N1	81.13 (8)	C11—C12—H12A	120.1
O6—Nd1—N1	121.37 (8)	C14—C13—C12	119.3 (3)
O9—Nd1—N1	102.84 (8)	C14—C13—H13A	120.4
O8—Nd1—N1	70.39 (8)	C12—C13—H13A	120.4
O5—Nd1—N1	77.07 (8)	C15—C14—C13	118.3 (3)
N3—Nd1—N1	124.02 (8)	C15—C14—H14A	120.8
O2—Nd1—N2	141.22 (8)	C13—C14—H14A	120.8
O3—Nd1—N2	143.39 (7)	N3—C15—C14	123.4 (3)
O6—Nd1—N2	115.13 (8)	N3—C15—H15A	118.3
O9—Nd1—N2	72.18 (8)	C14—C15—H15A	118.3
O8—Nd1—N2	89.48 (8)	O1—C16—C17	112.2 (3)
O5—Nd1—N2	75.82 (8)	O1—C16—H16A	109.2
N3—Nd1—N2	62.91 (8)	C17—C16—H16A	109.2
N1—Nd1—N2	62.43 (8)	O1—C16—H16B	109.2
O2—Nd1—N4	98.34 (8)	C17—C16—H16B	109.2
O3—Nd1—N4	88.77 (8)	H16A—C16—H16B	107.9
O6—Nd1—N4	24.74 (7)	C18—C17—C16	123.5 (4)
O9—Nd1—N4	142.86 (8)	C18—C17—H17A	118.2
O8—Nd1—N4	167.30 (8)	C16—C17—H17A	118.2
O5—Nd1—N4	25.20 (7)	C17—C18—H18A	120.0
N3—Nd1—N4	72.29 (8)	C17—C18—H18B	120.0
N1—Nd1—N4	100.72 (8)	H18A—C18—H18B	120.0
N2—Nd1—N4	94.35 (8)	C20—C19—C24	120.9 (3)
O2—Nd1—N5	79.81 (7)	C20—C19—H19A	119.6
O3—Nd1—N5	102.15 (8)	C24—C19—H19A	119.6
O6—Nd1—N5	151.74 (7)	C19—C20—C21	120.2 (3)
O9—Nd1—N5	24.95 (7)	C19—C20—H20A	119.9
O8—Nd1—N5	24.82 (7)	C21—C20—H20A	119.9
O5—Nd1—N5	155.25 (7)	C22—C21—C20	119.3 (3)
N3—Nd1—N5	95.51 (8)	C22—C21—H21A	120.3
N1—Nd1—N5	86.52 (8)	C20—C21—H21A	120.3
N2—Nd1—N5	80.20 (8)	C23—C22—C21	120.8 (3)
N4—Nd1—N5	167.79 (8)	C23—C22—H22A	119.6
C8—O1—C16	118.8 (3)	C21—C22—H22A	119.6
C25—O2—Nd1	136.2 (2)	C22—C23—C24	120.8 (3)
C27—O3—Nd1	134.1 (2)	C22—C23—H23A	119.6
N4—O5—Nd1	95.62 (19)	C24—C23—H23A	119.6
N4—O6—Nd1	97.14 (18)	C23—C24—C19	118.0 (3)
N5—O8—Nd1	97.23 (18)	C23—C24—C25	122.8 (3)
N5—O9—Nd1	97.11 (18)	C19—C24—C25	119.2 (3)
C1—N1—C5	117.8 (3)	O2—C25—C26	123.8 (3)
C1—N1—Nd1	120.7 (2)	O2—C25—C24	116.6 (3)
C5—N1—Nd1	121.33 (19)	C26—C25—C24	119.6 (3)
C6—N2—C10	117.6 (3)	C27—C26—C25	125.8 (3)
C6—N2—Nd1	120.74 (19)	C27—C26—H26	117.1
C10—N2—Nd1	120.26 (19)	C25—C26—H26	117.1
C15—N3—C11	118.3 (3)	O3—C27—C26	125.0 (3)
C15—N3—Nd1	119.7 (2)	O3—C27—C28	116.0 (3)
C11—N3—Nd1	121.7 (2)	C26—C27—C28	118.9 (3)
O4—N4—O6	121.9 (3)	C33—C28—C29	118.4 (3)
O4—N4—O5	121.6 (3)	C33—C28—C27	120.1 (3)
O6—N4—O5	116.5 (3)	C29—C28—C27	121.5 (3)
O4—N4—Nd1	169.6 (2)	C30—C29—C28	120.2 (3)
O6—N4—Nd1	58.12 (16)	C30—C29—H29A	119.9
O5—N4—Nd1	59.18 (17)	C28—C29—H29A	119.9
O7—N5—O8	122.7 (3)	C31—C30—C29	120.7 (4)
O7—N5—O9	121.4 (3)	C31—C30—H30A	119.7
O8—N5—O9	115.9 (3)	C29—C30—H30A	119.7
O7—N5—Nd1	179.3 (2)	C30—C31—C32	120.0 (3)
O8—N5—Nd1	57.95 (16)	C30—C31—H31A	120.0
O9—N5—Nd1	57.94 (15)	C32—C31—H31A	120.0
N1—C1—C2	124.2 (3)	C31—C32—C33	119.6 (3)
N1—C1—H1A	117.9	C31—C32—H32A	120.2
C2—C1—H1A	117.9	C33—C32—H32A	120.2
C1—C2—C3	118.2 (3)	C32—C33—C28	121.1 (3)
C1—C2—H2A	120.9	C32—C33—H33A	119.4
C3—C2—H2A	120.9	C28—C33—H33A	119.4
C4—C3—C2	118.6 (3)	N6—C34—C35	177.6 (5)
C4—C3—H3A	120.7	C34—C35—H35A	109.5
C2—C3—H3A	120.7	C34—C35—H35B	109.5
C3—C4—C5	120.6 (3)	H35A—C35—H35B	109.5
C3—C4—H4A	119.7	C34—C35—H35C	109.5
C5—C4—H4A	119.7	H35A—C35—H35C	109.5
N1—C5—C4	120.7 (3)	H35B—C35—H35C	109.5
